# A phase I study of the tyrosine kinase inhibitor anlotinib combined with platinum/pemetrexed-based chemotherapy in untreated nonsquamous non-small-cell lung cancer

**DOI:** 10.1007/s10637-021-01179-2

**Published:** 2021-11-01

**Authors:** Meijuan Huang, Yongmei Liu, Min Yu, Yanying Li, Yan Zhang, Jiang Zhu, Li Li, You Lu

**Affiliations:** 1 Department of Thoracic Oncology, Cancer Center and State Key Laboratory of Biotherapy, West China Hospital, Sichuan University, Chengdu, 610041 China; 2 Laboratory of Clinical Cell Therapy, West China Hospital, Sichuan University, Chengdu, 610041 China

**Keywords:** Anlotinib, Phase I study, Non-small-cell lung cancer, first-line therapy

## Abstract

*Background.* Anlotinib hydrochloride is an oral small molecule inhibitor of multiple tyrosine kinases, and it has been approved as a third-line therapy for patients with advanced non-small-cell lung cancer (NSCLC) in China. This dose-exploration study was designed to investigate the feasibility of anlotinib in combination with other chemotherapy regimens in patients with nonsquamous NSCLC. *Methods.* This phase I study followed a 3 + 3 dose reduction design with three doses of anlotinib (12 mg, 10 mg, and 8 mg). Anlotinib was given at an initial dose of 12 mg with pemetrexed (500 mg/m^2^) plus cisplatin (75 mg/m^2^) or carboplatin (AUC = 5) on 21-day cycles for 4 cycles. The primary goal of the study was to identify the maximum tolerated dose (MTD), and secondary endpoints included progression-free survival (PFS) and overall survival (OS). *Results.* A total of eight participants were enrolled. Dose-limiting toxicities (DLTs) were observed in two patients (pts) at anlotinib 12 mg (grade 3 hand-foot syndrome and grade 3 appetite loss). No DLTs occurred with 10 mg anlotinib, and the MTD was 10 mg. Among seven evaluable pts, four achieved a confirmed partial response (PR), and three had stable disease (SD). With a median follow-up of 10.05 months, the median PFS was 7.00 months (95% CI: 2.76 to NE). Grade 3 treatment-related adverse events (TRAEs) included appetite loss (n = 2), hypertension (n = 2), thrombocytopenia (n = 1), diarrhea (n = 1) and hand-foot syndrome (n = 1). No grade 4 or grade 5 TRAEs were observed during the treatment. *Conclusion.* The feasible dose of anlotinib in combination with platinum/pemetrexed-based chemotherapy as a first-line regimen was 10 mg, which was well tolerated and showed promising antitumor activity in advanced nonsquamous NSCLC.

## Introduction

In recent years, various antiangiogenic therapies have been evaluated in combination with platinum-based chemotherapy as a first-line treatment for advanced NSCLC without driver gene mutations [1, 2]. However, only bevacizumab, a humanized monoclonal antibody that binds vascular endothelial growth factor (VEGF), has been approved in the clinic [3–5]. Small molecule tyrosine kinase inhibitors (TKIs) with antiangiogenic activity, such as sorafenib, cediranib and vandetanib, have also been investigated in this setting, but no significant improvement in survival was observed. In addition, most of these oral TKIs result in added toxicities. Therefore, clinical trials involving antiangiogenic TKIs in combination with chemotherapy for untreated NSCLC are worthy of further exploration.

Anlotinib is an oral angiokinase inhibitor that blocks VEGFR 1 to 3, platelet-derived growth factor receptor (PDGFR) α and β, and fibroblast growth factor receptor (FGFR) 1 to 4 [6, 7]. In addition, the receptor kinases RET, c-Kit and c-Met are also inhibited. With a low half maximal inhibitory concentration (IC_50_), anlotinib has shown promising antitumor activity in various tumors, including NSCLC, in previous studies [6, 8], and it is the only CFDA-approved angiokinase inhibitor for advanced NSCLC [9]. Based on the pharmacokinetics of anlotinib, limited drug-drug interactions allow its combination with cytotoxic drugs. Therefore, an anlotinib-platinum-pemetrexed (APP) regimen was investigated in this trial. Considering the toxicity profile of anlotinib monotherapy as well as the predicted intolerance of the combination therapy, a dose-reduction design was employed, reducing the administered dose of anlotinib from 12 to 10 mg and 8 mg.

## Patients and methods

### Study population

Eligible patients had histologically or cytologically confirmed stage IIIB/IIIC/IV nonsquamous NSCLC; were negative for mutations of EGFR\ALK\ROS1; were aged 18 to 70; never received any systematic treatment (including immunotherapy); had an Eastern Cooperative Oncology Group (ECOG) performance status 0–1; had an expected survival time ≥ 3 months; and presented no major organ dysfunction. Patients were excluded if they had active brain metastases, uncontrolled hypertension, severe cardiovascular diseases, or coagulation abnormalities.

This study was reviewed and approved by the Institutional Review Board of West China Hospital, Sichuan University. Written informed consent was obtained from all participants. This study is registered with ClinicalTrials.gov, number NCT04012619.

### Study design

This study employed a standard 3 + 3 dose reduction design, and eligible patients received an anlotinib chemotherapy regimen after a 21-day cycle for 4 cycles. According to the ALTER0303 study, the initial dose of anlotinib was set as 12 mg/day with a 2-week on/1-week off schedule. The dose was reduced to 10 mg/day and 8 mg/day in sequence depending on observed DLTs in cycle 1. If there were no DLTs, the dose of anlotinib in the combined chemotherapy regimen was determined to be 12 mg/day. If a DLT occurred in ≥ 2 of 3 enrolled subjects, the initial dose was reduced to 10 mg/day. If DLT occurred in 1 of 3 subjects, the dose level was followed up, and 3 additional subjects were enrolled. If 1 DLT occurred in the last 3 subjects, the dose was reduced to 10 mg/day. Pemetrexed (500 mg/m^2^) and either cisplatin (75 mg/m^2^) or carboplatin (AUC = 5) were intravenously given on Day 1 of each cycle. Patients who had disease control after the combination regimen continued to receive anlotinib maintenance until disease progression was observed.

### Assessments

Adverse events (AEs) were monitored during the study and summarized according to the National Cancer Institute Common Terminology Criteria for Adverse Events (NCI CTCAE) version 4.0. Based on Response Evaluation Criteria In Solid Tumors (RECIST) version 1.1, the tumor response was evaluated by computerized tomography (CT) or magnetic resonance imaging (MRI) at baseline and subsequently every 6 weeks until study termination. Patients who achieved a complete response (CR) or partial response (PR) were required to have efficacy confirmation at least 4 weeks after the initial evaluation.

The primary endpoint was the MTD of anlotinib, at which less than 33% of patients experienced a DLT in the first treatment cycle. A DLT involving hematological toxicity was defined as grade 4 and above, non-hematological toxicity as grade 3 and above, and liver and kidney function injury as grade 2 and above. The secondary endpoints included PFS and OS. PFS was defined as the time from the date of randomization to the date of disease progression or death. OS was defined as the time from the date of randomization to the date of death.

### Statistical analysis

The recruitment of a minimum of 3 patients and a maximum of 18 patients was planned in accordance with the 3 + 3 study design. Analyses were based on an Apr 10, 2020, database lock. All patients who received at least one dose of the investigational drug were included in the safety assessment, and those who completed at least one cycle of the treatment were eligible for efficacy evaluation. The baseline demographic characteristics and frequency of adverse events are summarized with descriptive statistics. Two-sided 95% exact CIs were calculated for the ORR and DCR using the Clopper-Pearson method, and estimated time-to-event endpoints were calculated with the Kaplan–Meier method with two-sided 95% CIs for medians. All statistical analyses were carried out with SAS 9.1.3 software.

## Results

### Demographic characteristics

A total of 8 patients were enrolled in this trial between April 2019 and November 2019. All patients were untreated before enrollment and had histologically confirmed adenocarcinoma without sensitizing EGFR/ALK/ROS1 alterations (Table 1). The median age was 62 years (39.0–69.0), and 7 of them had an ECOG performance status of 1.

**Table 1:** Patient characteristics at baseline

Characteristics	Anlotinib (n = 8)
Age, median (range), years	62.0 (39.0–69.0)
Gender, n (%)	
Male	5 (62.5)
Female	3 (37.5)
ECOG, n (%)	
0	1 (12.5)
1	7 (87.5)
Stage, n (%)	
IVA	5 (62.5)
IVB	3 (37.5)
Smoking history, n (%)	
Ever	4 (50.0)
Never	4 (50.0)

### DLT and MTD

In the study, there were 4 patients in the 12 mg dose group and 4 patients in the 10 mg dose group. Two DLTs were observed in two pts separately at anlotinib 12 mg (grade 3 hand-foot syndrome and grade 3 appetite loss). The patient with appetite loss stopped anlotinib; the patient with hand-foot syndrome reduced their anlotinib dose. Another patient with grade 3 hypertension and grade 2 proteinuria resumed anlotinib after suspension.

No DLTs occurred at 10 mg. Only 1 patient treated with 10 mg anlotinib experienced grade 3 TREA (thrombocytopenia); therefore, the MTD was determined to be 10 mg, at which less than 33% of pts experienced a DLT when treated with the anlotinib-platinum-pemetrexed combination.

### Safety

AEs were monitored throughout the treatment until 30 days after termination of the trial. AEs occurred in all patients, and 5 of them experienced grade 3 AEs (Table 2). The most frequent AEs were appetite loss (87.5%), alanine transaminase elevation (62.5%), aspartate transaminase elevation (62.5%), thrombocytopenia (50%), nausea (50%), hypertension (50%) and fatigue (50%). Grade 3 treatment-related adverse events (TRAEs) included appetite loss (n = 2), hypertension (n = 2), thrombocytopenia (n = 1), diarrhea (n = 1) and hand-foot syndrome (n = 1) (Table 3). No grade 4 or grade 5 TRAEs were observed during the treatment.

**Table 2:** Overview of adverse events and discontinuations

n (%)	Anlotinib (n = 8)
Total patients with ≥ 1 AE	8 (100.0)
Serious AE	3 (37.5)
Severe AE (grade ≥ 3)	5 (62.5)
AE leading to death	0 (0.0)
AE leading to withdrawal from treatment	2 (25.0)
AE leading to dose modification/interruption	5 (62.5)
Treatment-related AE	8 (100.0)
Treatment-related serious AE	3 (37.5)

**Table 3:** Summary of most common TRAEs reported in ≥ 20% of patients

		**AE, n (%)**				** ≥ Grade III AE**	
	**12 mg (n = 4)**	**10 mg (n = 4)**	**Total**	**12 mg (n = 4)**	**10 mg (n = 4)**		**Total**
TRAEs	4 (100.0)	4 (100.0)	8 (100.0)	4 (100.0)	1 (25.0)		5 (62.5)
Appetite down	4 (100.0)	3 (75.0)	7 (87.5)	0 (0.0)	0 (0.0)		2 (25.0)
Hypertriglyceridemia	2 (50.0)	3 (75.0)	5 (62.5)	0 (0.0)	0 (0.0)		0 (0.0)
Alanine transaminase elevation	3 (75.0)	2 (50.0)	5 (62.5)	0 (0.0)	0 (0.0)		0 (0.0)
Aspartate transaminase elevation	2 (50.0)	3 (75.0)	5 (62.5)	0 (0.0)	0 (0.0)		0 (0.0)
Hypertension	3 (75.0)	1 (25.0)	4 (50.0)	2 (50.0)	0 (0.0)		2 (25.0)
Thrombocytopenia	2 (50.0)	2 (50.0)	4 (50.0)	0 (0.0)	1 (25.0)		1 (12.5)
Nausea	1 (25.0)	3 (75.0)	4 (50.0)	0 (0.0)	0 (0.0)		0 (0.0)
Fatigue	2 (50.0)	2 (50.0)	4 (50.0)	0 (0.0)	0 (0.0)		0 (0.0)
Neutropenia	1 (25.0)	2 (50.0)	3 (37.5)	0 (0.0)	0 (0.0)		0 (0.0)
Hypercholesterolemia	1 (25.0)	2 (50.0)	3 (37.5)	0 (0.0)	0 (0.0)		0 (0.0)
Leukopenia	0 (0.0)	3 (75.0)	3 (37.5)	0 (0.0)	0 (0.0)		0 (0.0)
Dizziness	1 (25.0)	2 (50.0)	3 (37.5)	0 (0.0)	0 (0.0)		0 (0.0)
Hand-foot syndrome	2 (50.0)	0 (0.0)	2 (25.0)	1 (25.0)	0 (0.0)		1 (12.5)
Diarrhea	2 (50.0)	0 (0.0)	2 (25.0)	1 (25.0)	0 (0.0)		1 (12.5)
Vomiting	2 (50.0)	0 (0.0)	2 (25.0)	0 (0.0)	0 (0.0)		0 (0.0)
Gum pain	1 (25.0)	1 (25.0)	2 (25.0)	0 (0.0)	0 (0.0)		0 (0.0)
Proteinuria	1 (25.0)	1 (25.0)	2 (25.0)	0 (0.0)	0 (0.0)		0 (0.0)
Hypothyroidism	1 (25.0)	1 (25.0)	2 (25.0)	0 (0.0)	0 (0.0)		0 (0.0)
Anemia	0 (0.0)	2 (50.0)	2 (25.0)	0 (0.0)	0 (0.0)		0 (0.0)
Hoarseness	1 (25.0)	1 (25.0)	2 (25.0)	0 (0.0)	0 (0.0)		0 (0.0)
Thyroid stimulating hormone elevation	2 (50.0)	0 (0.0)	2 (25.0)	0 (0.0)	0 (0.0)		0 (0.0)

*AE* adverse event, *TRAEs* treatment-related adverse events

### Efficacy

**Fig. 1:**
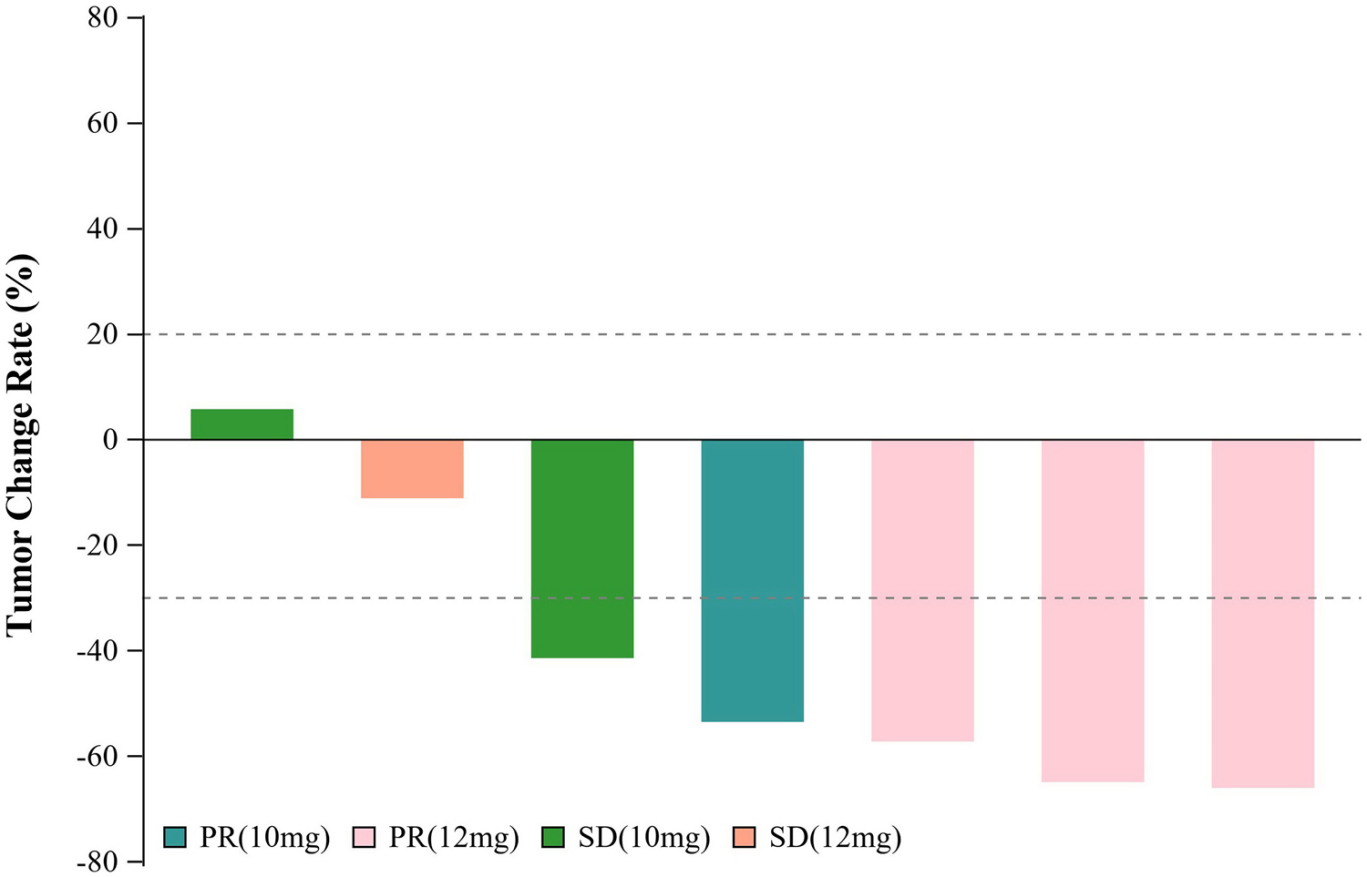
Best change from baseline in sum of longest target lesion diameter per patient

The tumor response was assessed in 7 cases: 57.14% of cases had a confirmed objective response (95% CI: 18.41%, 90.10%) and 100% had confirmed disease control (95% CI: 59.04%, 100.00%). One patient withdrew consent before completing the first cycle. Among the evaluable cases, 6 exhibited tumor shrinkage (Fig. 1). Four (57.14%) had a confirmed PR, 3 (42.86%) had SD, and no PD was reported. At the data cutoff on Apr 10, 2020, six pts had terminated the treatment, predominantly due to disease progression. With a median follow-up of 10.05 months (95% CI: 5.59, 11.40), the median PFS time was 7.00 months (95% CI: 2.76 to NE).

**Fig. 2:**
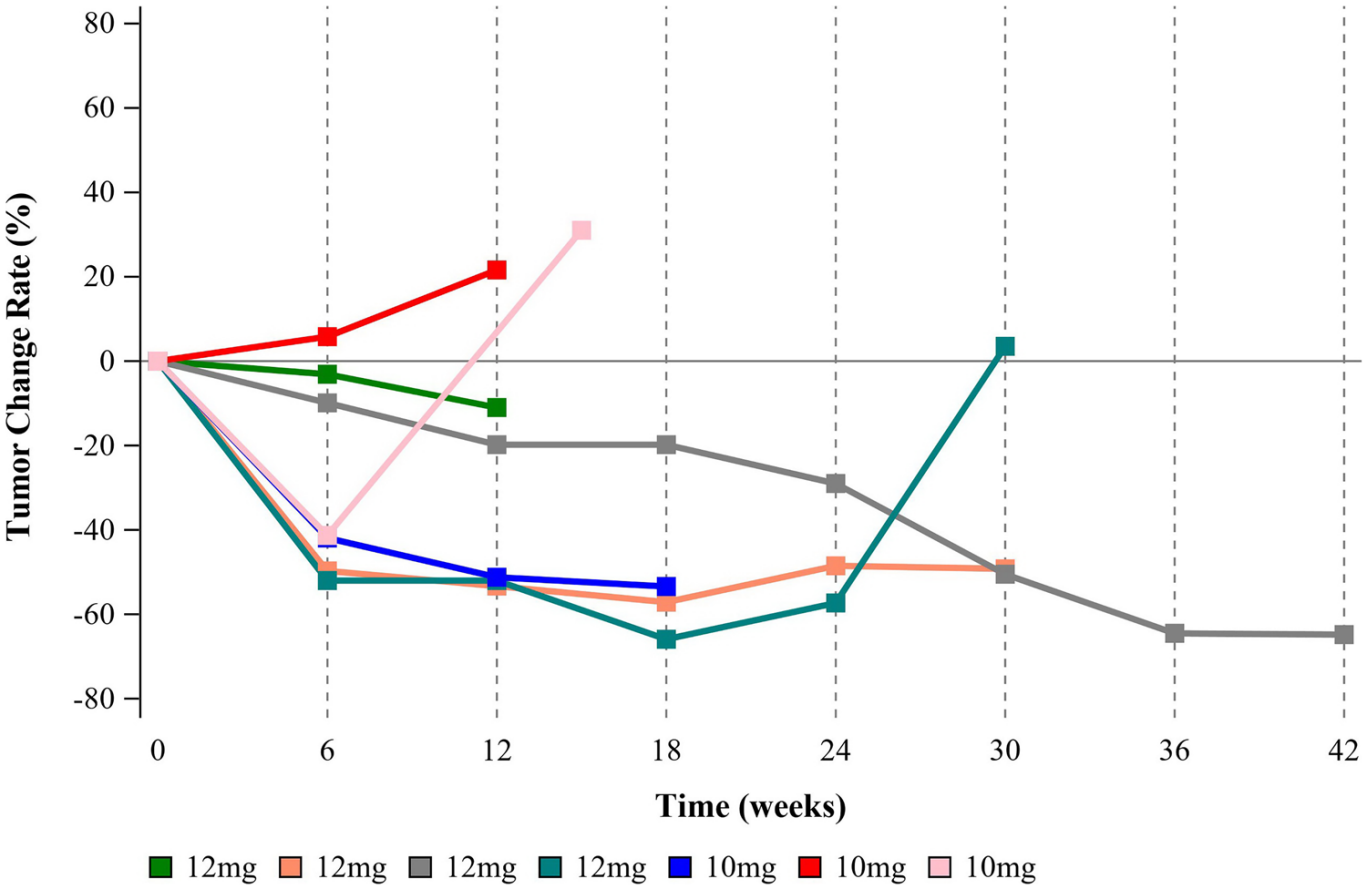
The change curve with time in sum of longest target lesion diameter per patient

Of the four responders, three were in the 12 mg group, and another was in the 10 mg group (Fig. 1). The responder who experienced the maximum tumor shrinkage was in the high-dose group with a response by the first scan (Fig. 2). At the time of the analysis, one subject in the 12 mg group was still in this trial and showed sustained remission after 4 cycles of the combination treatment and 10 cycles of anlotinib maintenance (Fig. 2).

## Discussions

The tolerability, safety and efficacy of anlotinib combined with chemotherapy were explored in this study, and it was demonstrated that the anlotinib-platinum-pemetrexed regimen was well tolerated in advanced NSCLC patients at an anlotinib dose of 10 mg. Moreover, the combination therapy demonstrated promising antitumor activity in these patients with an acceptable safety profile.

Overall, AEs in our study were mild, and the majority were grade 1 or 2. Specific adverse events related to anlotinib, such as hypertension and hand-foot syndrome, were in the range of incidence reported in previous trials involving anlotinib [10]. On the other hand, the combination therapy resulted in a higher occurrence of hematological toxicities such as neutropenia, thrombocytopenia, leukopenia and anemia than that in response to platinum-pemetrexed alone, indicating an additive myelosuppressive effect of anlotinib, which has been mentioned by other investigators [11]. However, the hematological events significantly declined in the 10 mg group, and the majority of these events were fully resolved. Overall, there was no evidence that anlotinib potentiated chemotherapy toxicity, and each individual drug could be administered in combination at the recommended dosage.

Remarkably, the tumor response of anlotinib-platinum-pemetrexed in this trial (57.14%) was encouraging, since pemetrexed-cisplatin alone only showed a response rate of 30.6% in the JMDB study, indicating a synergistic effect of anlotinib. Furthermore, we reported a median PFS of 7.00 months, and to the best of our knowledge, this is the first reported PFS of the APP regimen for treatment-naïve NSCLC. These favorable results were mainly attributed to the high selectivity and potent inhibition of VEGFR2 as well as broad inhibitory effects of anlotinib on other proangiogenic pathways [12, 13]. In addition to its antiangiogenic activity, anlotinib suppresses tumor cell growth and migration by blocking c-Kit, c-Met and RET [14]. Collectively, our findings suggested that the combined treatment was efficacious and could be an alternative option for nonsquamous NSCLC.

Limitations to the findings included the small size of the cohort, which makes it difficult to draw firm conclusions on the antitumor efficacy of this regimen. In addition, the single-arm design of the trial limited its ability to directly compare results with those in response to chemotherapy alone. Considering these findings, the APP regimen could be further validated by controlled trials in the future.

## Data Availability

The datasets used or analyzed during the current study are available from the corresponding author on reasonable request.
